# Phase 1/2 study of pacritinib, a next generation JAK2/FLT3 inhibitor, in myelofibrosis or other myeloid malignancies

**DOI:** 10.1186/s13045-016-0367-x

**Published:** 2016-12-08

**Authors:** Srdan Verstovsek, Olatoyosi Odenike, Jack W. Singer, Tanya Granston, Suliman Al-Fayoumi, H. Joachim Deeg

**Affiliations:** 1 Department of Leukemia, University of Texas MD Anderson Cancer Center, 1515 Holcombe Blvd, Unit 428, Houston, TX 77030 USA; 2 Hematology/Oncology, University of Chicago Medical Center, Chicago, IL USA; 3 CTI BioPharma Corp., Seattle, WA USA; 4 Fred Hutchinson Cancer Research Center and University of Washington, Seattle, WA USA

**Keywords:** FMS-like tyrosine kinase 3 inhibitors, Janus kinase 2 inhibitors, Myelofibrosis, Myeloid malignancies, Myelosuppression, Pacritinib, Pharmacokinetics, Quality of Life, Splenomegaly

## Abstract

**Background:**

Pacritinib (SB1518) is a highly selective kinase inhibitor with specificity for JAK2, FLT3, IRAK1, and CFS1R. This multicenter phase 1/2 study evaluated the maximum tolerated dose (MTD), safety, and clinical activity of pacritinib in patients with myelofibrosis (MF) and other advanced myeloid malignancies.

**Methods:**

In the phase 1 dose-escalation part of the study, 43 adults with advanced myeloid malignancies received pacritinib 100 to 600 mg once daily (QD). In the phase 2 part of the study, 31 adults with refractory or intermediate- or high-risk newly diagnosed MF and any degree of cytopenia received pacritinib 400 mg QD. The primary endpoint is a ≥35% reduction in spleen volume at week 24 as determined by magnetic resonance imaging.

**Results:**

Five patients (11.6%) experienced a dose-limiting toxicity during cycle 1 of phase 1. The clinical benefit rate was 86.0% (13 patients achieving clinical improvement and 24 patients having stable disease). The MTD was established at 500 mg QD, and the recommended phase 2 dose was 400 mg QD. In phase 2, the primary endpoint was achieved by 23.5% of evaluable patients (4/17), with 47.4% (9/19) achieving a ≥50% spleen length reduction at week 24 as measured by physical examination. At week 24, 38.9% of evaluable patients (7/18) achieved a ≥50% decrease in MF Quality of Life and Symptom Assessment total score. Gastrointestinal toxicities were the most common adverse events and were predominantly grade 1/2 in severity. Grade 3/4 anemia was reported in 5/31 patients and grade 3/4 thrombocytopenia was reported in 3/31 patients. The most frequent AEs considered to be treatment related were diarrhea (28/31), nausea (15/31), vomiting (9/31), and fatigue (4/31). Grade 3 treatment-related AEs were reported in seven patients (22.6%), four of whom had diarrhea. No grade 4/5 treatment-related AEs were reported. No leukopenia, neutropenia, or lymphopenia were reported.

**Conclusions:**

Pacritinib was well tolerated and demonstrated clinical activity in MF. The study suggests that pacritinib has unique characteristics, namely a lack of substantial myelosuppression and manageable side effects, making it an attractive target for further evaluation in MF.

**Trial registration:**

Retrospectively registered at www.clinicaltrials.gov (#NCT00719836) on July 20, 2008.

**Electronic supplementary material:**

The online version of this article (doi:10.1186/s13045-016-0367-x) contains supplementary material, which is available to authorized users.

## Background

Aberrant activation of the JAK/STAT pathway plays a central role in myeloproliferative neoplasms (MPNs), a group of heterogeneous diseases including polycythemia vera (PV), essential thrombocythemia (ET), and chronic idiopathic myelofibrosis (MF) [1, 2]. The discovery of a dominant gain-of-function mutation in the *JAK2* gene (*JAK2*V617F) in most patients with PV, and 30–60% of patients with ET or MF allowed identification of JAK2 as a new therapeutic target for these disorders [3–6]. MPN patients negative for *JAK2*V617F may display mutations in the thrombopoietin receptor gene (*MPL*) [7] or the calreticulin gene (*CALR*) [8], which may also lead to dysregulated JAK/STAT-dependent processes. Greater understanding of the JAK/STAT pathway and its role in MPNs has led to the development of therapeutic JAK inhibitors. These agents are not mutation specific and are effective in MPN patients with and without the *JAK2* mutation [9].

FMS-like tyrosine kinase 3 (*FLT3*) mutations that involve internal tandem duplications of the juxtamembrane domain-coding sequence are found in 20–30% of patients with acute myeloid leukemia (AML), typically in de novo AML, and are associated with poor clinical outcomes [10–12]. Upregulation of FLT3 may also be involved in driving the abnormal production of peripheral platelets, characteristic of MF [13].

Pacritinib (SB1518) is a kinase inhibitor with specificity for JAK2, FLT3, IRAK1, and CFS1R [14–16]; it does not have specificity for JAK1 at pharmacologically-relevant levels [14]. Pacritinib demonstrated a favorable safety and pharmacological activity profile in preclinical hematologic malignancy models [17, 18]. The objectives of this multicenter phase 1/2 study were to determine the maximum tolerated dose (MTD), pharmacokinetics, safety, and clinical activity of pacritinib in patients with MF and other advanced myeloid malignancies.

## Methods

This clinical study included a phase 1 dose escalation component to establish the MTD of pacritinib in patients with advanced myeloid malignancies, followed by an open-label, single-arm phase 2 component to assess the clinical activity (based on spleen response rate) and tolerability of pacritinib administered at the recommended dose in patients with MF.

### Ethics, consent, and data analysis

The study was conducted at three centers in the USA between June 2008 and October 2011 in accordance with the Declaration of Helsinki, International Conference on Harmonization guidelines, and relevant laws and regulations. The protocol was approved by the institutional review boards at each study site. Written informed consent was obtained from all patients prior to participation. Data collection, data entry, and database lock were performed by S*BIO Pte Ltd (the original study sponsor) and data analysis was performed by CTI BioPharma Corp. (who acquired pacritinib in 2012). All authors had access to primary clinical trial data.

### Patients

Patients were eligible for inclusion in phase 1 if they had a histologically confirmed myeloid malignancy and had failed standard therapies or were not candidates for palliative therapies. Eligible malignancies included AML; chronic myeloid leukemia (CML) in accelerated or blast phase; high-risk myelodysplastic syndrome (refractory anemia with 5–19% bone marrow blasts or chronic myelomonocytic leukemia with >5% bone marrow blasts); and advanced MF with ≥1 poor prognostic feature (hemoglobin <10 g/dL, platelets <100 × 10^9^/L, white blood cells <4 or >30 × 10^9^/L, or symptomatic splenomegaly ≥10 cm below the left costal margin). Patients were eligible for inclusion in phase 2 if they had MF (including post-ET [PET]-/post-PV [PPV]-MF) that had relapsed or was not well controlled with standard therapy and had splenomegaly ≥5 cm below the costal margin. Patients were also eligible if they had newly diagnosed MF classified as intermediate- or high-risk [19] and were not suitable for standard therapy. In both components of the study, patients were required to be aged ≥18 years with Eastern Cooperative Oncology Group performance status (ECOG PS) 0–2; adequate liver and renal function; corrected QTc interval ≤0.47 s (Bazett formula); a 2-week window from prior disease-directed therapy with recovery from prior toxicities to grade ≤1; and a 1-week window from prior hydroxyurea and CYP3A4 inducer/inhibitor treatment. Patients with uncontrolled intercurrent illness, concurrent malignancy, HIV, or active hepatitis A, B, or C were excluded, as were those who were pregnant, lactating, or unwilling/unable to undergo a magnetic resonance imaging (MRI) scan (phase 2 only).

### Study design and treatment

Phase 1 was a 3 + 3 dose-escalation study. Three to six patients were enrolled at each dose level, depending upon observed dose-limiting toxicities (DLTs) in the cohort. Each patient participated in only one cohort. Oral pacritinib was administered to the first cohort at 100 mg once daily (QD) for 25 consecutive days in a 28-day cycle in cycle 1, with 3 days of rest for pharmacokinetic (PK) sampling, followed by continuous 28-day dosing for the remaining cycles. Subsequent cohorts received escalating pacritinib dose levels of 150, 200, 300, 400, 500, and 600 mg QD. All patients at each dose level were observed through the end of the first cycle before the next dose level could begin treatment.

Each patient enrolled in phase 1 was allowed two dose escalations at the investigator’s discretion and with the sponsor’s approval. Those who had completed ≥3 treatment cycles without disease progression could escalate to the last dose level cleared as tolerable. Once the MTD was reached, patients treated at lower dose levels (including those who had undergone one previous dose escalation) were permitted to escalate to the recommended dose.

A DLT was defined as a grade 3/4 non-hematologic adverse event (AE), a clinically significant grade 4 hematologic AE that persisted >28 days in the absence of active hematologic malignancy with bone marrow involvement, or grade 4 febrile neutropenia/granulocytopenia during cycle 1 that was possibly, probably, or definitely related to the study drug. In addition, any other clinically significant events that resulted in study drug interruption and/or dose reduction could be considered a DLT. Grade 3 diarrhea or vomiting were only considered DLTs if they persisted for >7 days despite treatment. Fatigue was only considered a DLT if it was grade 4 and persisted for >2 weeks. In patients with grade 2 alanine aminotransferase/aspartate aminotransferase elevations at baseline, elevations were only considered DLTs if they exceeded three times the baseline level and were confirmed 1 week later. Recurrence of prior grade 3 hyperuricemia was not considered a DLT.

Phase 2 dose selection was based on exposure, safety, pharmacodynamic, and clinical benefit data from phase 1. In phase 2, patients received pacritinib 400 mg QD without interruption in a 28-day cycle.

Treatment was able to be withheld for up to 2 weeks for treatment-emergent AEs. After toxicity resolution, patients could resume treatment at the same or a reduced dose at the discretion of the investigator after discussion with the sponsor (Additional file 1: Table S1). Up to two dose reductions were allowed; however, re-escalation was not permitted.

Two forms of pacritinib were developed. The study was initiated with the hydrochloride (HCl) salt formulation, which was also used for non-clinical and early clinical development. A citrate salt formulation containing the same active moiety was later designed to improve the physical stability of the product. Six new patients were enrolled in phase 1 and received pacritinib citrate salt 200 mg QD. Patients enrolled in phase 1 were subsequently switched to the citrate salt and all patients enrolled in phase 2 received the citrate salt.

### Study procedures

At screening/baseline, patients underwent a medical history, physical examination, vital sign assessment, ECOG PS assessment, electrocardiogram, and assessment of hematologic parameters and blood chemistry. Hematology/blood chemistry, physical examinations, and vital sign assessments were performed throughout treatment. Toxicity was assessed using the National Cancer Institute Common Terminology Criteria for Adverse Events (NCIC-CTCAE), version 3.0, with relationship to treatment assigned by the investigator and were recorded from the first dose of pacritinib until 30 days after the last dose. Bone marrow biopsy and/or aspirate samples were drawn at baseline, on day 1 of cycle 4 and cycle 10, as clinically indicated, and at the termination visit. Disease response and progression were determined using the relevant International Working Group (IWG) criteria [20–23].

In phase 1, full PK sampling was performed on days 1 and 15 of cycle 1 and day 1 of cycle 2. Trough PK samples were drawn prior to dosing on days 8 and 22 of cycle 1 and at 24, 48, and 72 h after dosing on day 25 of cycle 1. A sample was collected before dosing on day 1 of cycles 4, 7, and 10. Pacritinib concentrations were determined using a validated liquid chromatography/tandem mass spectrometry method. An analysis of clinical exposures from the HCl and citrate salts of pacritinib was performed comparing Day 1 PK data from 12 patients who received the HCl salt formulation in the 200 mg cohorts of this study and two other pacritinib studies [NCT00741871 and NCT00745550]) with six patients in the 200 mg cohort of this study who received the citrate salt formulation.

In phase 2, abdominal MRI to assess spleen and liver volume was performed on day 1 of cycles 1, 4, and 7 and then day 1 of every third cycle through cycle 25. Spleen size (length) was also assessed by manual palpation with each scheduled physical examination. The phase 2 study protocol was amended in September 2009 to include the MF Quality of Life and Symptoms Assessment Tool. The tool was derived from the MF Symptom Assessment Form [24] and rated disease-related symptoms (worst fatigue, early satiety, abdominal pain or discomfort, night sweats, itching, and bone pain) and overall quality of life on a scale of 0 (absent) to 10 (worst imaginable). The instrument was administered pre-dose on day 1 of cycles 1, 2, 4, 7, and 10 and then on day 1 of every third cycle through cycle 25.

### Statistical analysis

The primary objective of phase 1 was to establish the MTD of pacritinib in patients with advanced myeloid malignancies, defined as the highest dose level at which no more than one out of six patients in the cohort experienced a DLT during cycle 1 (25 days) of therapy. The DLT-evaluable population included all patients who completed at least 18 of the 25 originally assigned doses in cycle 1, or those who did not complete the required number of assigned doses and had a DLT. Secondary objectives in phase 1 of the study included safety and PK analysis. Clinical benefit rate was assessed as an exploratory endpoint, defined as the proportion of patients who experienced a complete response, partial response, clinical improvement, or stable disease (SD) during the study.

The primary endpoint of the phase 2 component was the proportion of the efficacy population who achieved a ≥35% reduction in spleen volume from baseline at week 24, as measured by MRI. Spleen response rate was also assessed in terms of a ≥35% reduction in spleen volume by MRI up to week 24 and end of treatment and as a ≥50% reduction in spleen length by physical examination up to week 24 and end of treatment. Duration of response was not analyzed due to limitations in the proportion of responders. An exploratory endpoint was the proportion of patients with a ≥50% reduction in MF Quality of Life and Symptoms Assessment Tool at week 24 compared with baseline.

The planned sample size of 29 patients for the phase 2 component provided 80% power to detect a spleen response rate of ≥30% versus the null hypothesis of a response rate of ≤10%, with *α* = 0.05 (two-sided). The efficacy-evaluable population included those patients with a baseline and corresponding follow-up value. The safety population included all enrolled patients who received ≥1 dose of pacritinib.

## Results

### Phase 1

#### Patients

In phase 1, 43 patients with advanced myeloid malignancies (MF: *n* = 36; AML: *n* = 7) were treated with pacritinib at doses ranging from 100 to 600 mg QD. Subject demographics and baseline characteristics are shown in Table 1. 

The majority of patients had splenomegaly (*n* = 28; 65.1%). At baseline, grade 2 or higher anemia and thrombocytopenia were reported in 28 (65.1%) and 21 (48.8%) patients, respectively. Of the 43 patients, 18 (41.9%) completed the study and 25 (58.1%) discontinued the study, primarily because of disease progression (*n* = 9) and the sponsor’s decision to terminate the study for financial reasons (*n* = 7).

**Table 1:** Baseline characteristics and demographics for patients enrolled in phase 1 (*n* = 43)

	Pacritinib							
	100 mg	150 mg	200 mg	300 mg	400 mg	500 mg	600 mg	All patients^a^
	*n* = 3	*n* = 6	*n* = 9	*n* = 6	*n* = 6	*n* = 7	*n* = 6	*n* = 43
Median age, years (range)	72.0 (59–78)	76.0 (66–86)	69.0 (63–76)	64.5 (50–77)	67.5 (52–83)	65.0 (49–77)	74.5 (53–76)	71.0 (49–86)
Male, *n* (%)	2 (66.7)	5 (83.3)	7 (77.8)	3 (50.0)	2 (33.3)	4 (57.1)	4 (66.7)	27 (62.8)
ECOG PS, *n* (%)								
0	0	2 (33.3)	5 (55.6)	4 (66.7%)	3 (50.0)	4 (57.1)	0	18 (41.9)
1	2 (66.7)	2 (33.3)	4 (44.4)	1 (16.7)	2 (33.3)	2 (28.6)	5 (83.3)	18 (41.9)
2	1 (33.3)	2 (33.3)	0	1 (16.7)	1 (16.7)	1 (14.3)	1 (16.7)	7 (16.3)
Median disease duration, months (range)	5.0 (4.1–6.7)	25.8 (8.6–174.4)	45.9 (2.3–410.2)	37.4 (8.5–341.8)	27.4 (5.1–132.2)	36.9 (10.0–182.9)	99.2 (13.7–196.9)	36.9 (2.3–410.2)
Current malignancy type, *n* (%)								
AML	0	3 (50.0)	0	0	3 (50.0)	0	1 (16.7)	7 (16.3)
*JAK2* mutation^b^, *n*/*N* (%)	–	1/3 (33.3)	–	–	2/3 (66.7)	–	1/1 (100)	4/7 (57.1)
Myelofibrosis	3 (100.0)	3 (50.0)	9 (100.0)	6 (100.0)	3 (50.0)	7 (100.0)	5 (83.3)	36 (83.7)
*JAK2* mutation^b^, *n*/*N* (%)	2/3 (66.7)	2/3 (66.7)	8/9 (88.9)	5/6 (83.3)	3/3 (100)	5/7 (71.4)	4/5 (80.0)	29/36 (80.6)
Median prior systemic therapies, *n* (range)	1.0 (0–4)	3.0 (2–4)	1.0 (0–4)	2.0 (1–4)	2.0 (1–4)	2.0 (0–4)	3.0 (1–4)	3.0 (0–4)
Median time since last cancer treatment, months (range)	2.4 (1.5–3.2)	1.2 (0.1–6.3)	2.0 (0.5–39.6)	8.0 (0.5–37.1)	1.5 (0.5–2.4)	2.8 (0.7–27.1)	6.3 (0.6–21.7)	2.2 (0.1–39.6)

^a^86% of patients were white

^b^All *JAK2* mutations were V617F

*AML* acute myeloid leukemia, *ECOG PS* Eastern Cooperative Oncology Group Performance Status, *MDS* myelodysplastic syndrome

#### DLTs and MTD

All 43 patients enrolled in phase 1 were evaluable for DLT assessment. Overall, five patients (11.6%) experienced a DLT during cycle 1 (Table 2). Diarrhea that was considered to be a DLT was reported in one patient at each of the following dose levels: 300, 500, and 600 mg. One patient in the 150 mg cohort experienced grade 3 QTc prolongation (QTc of 460 ms at screening, 491 ms on cycle 1 day 1, 504 ms on cycle 1 day 15, and 519 ms on cycle 1 day 22 [last assessment available]). The patient had baseline atrial fibrillation, a history of inferior myocardial infarction and right bundle branch block, hypokalemia and was receiving amiodarone, solifenacin, and fluconazole. Pacritinib was discontinued and the QTc prolongation resolved. One patient in the pacritinib 600 mg cohort experienced six distinct grade 1 or 2 events that were individually assessed as not serious but were considered as DLTs when taken together (Table 2). Relevant prior history included prostate cancer, renal cell carcinoma-post partial nephrectomy, cataract surgery, celiac sprue, and vertigo. Pacritinib was interrupted and the dosage was reduced to 400 mg QD. Nausea and vomiting resolved with antiemetic therapy. Dizziness and the decrease in performance status resolved; however, blurred vision and gait disturbance were ongoing at the end of study.

**Table 2:** Dose-limiting toxicities in phase 1 (*n* = 43)

Pacitinib dose cohort	Patients, *n*	DLTs in first cycle		
		*N* (%)	Description	Outcome
100 mg	3	0		
150 mg	6	1 (16.7)	Grade 3 ECG QT prolonged from days 18–47	Resolved following pacritinib discontinuation.
200 mg	9^a^	0		
300 mg	6	1 (16.7)	Grade 3 diarrhea on days 3–4.	Resolved following interruption of pacritinib and treatment with antidiarrheal medication. Pacritinib was restarted.
400 mg	6	0		
500 mg	7	1 (14.3)	Grade 2 diarrhea on days 8–140.	Resolved following dose reduction to 400 mg and treatment with antidiarrheal medication.
600 mg	6	2 (33.3)	Grade 3 diarrhea beginning on day 11.	Pacritinib was interrupted then reduced to 400 mg. Antidiarrheal medication was started. Diarrhea was ongoing at the end of treatment.
			Grade 1 nausea; grade 1 vomiting; grade 2 dizziness, grade 2 gait disturbance, grade 2 performance status decreased, grade 2 vision blurred	Pacritinib was interrupted then reduced to 400 mg. Nausea and vomiting resolved with antiemetic therapy after ~85 days; dizziness resolved after 49 days; performance status decrease resolved after 4 days. Gait disturbance and vision blurred were ongoing at the end of treatment.

^a^This cohort included three patients treated with the pacritinib HCl salt formulation and an additional six patients treated with the citrate salt formulation. The two salt forms have been shown to be pharmaceutically equivalent

*DLT* dose-limiting toxicity, *ECG* electrocardiogram

The MTD of pacritinib was determined to be 500 mg QD. Although only one of seven patients in the 500 mg QD cohort experienced a DLT, two discontinued treatment due to AEs, three required dose interruptions, three required dose reductions, and three experienced a serious AE (SAE). Therefore, pacritinib 400 mg QD was selected as the recommended phase 2 dose.

#### Safety

Table 3 summarizes treatment-emergent AEs occurring in ≥10% of patients in phase 1. The most frequent AEs were gastrointestinal in nature and were mainly grades 1 or 2 in severity. There were no discernible trends observed across dose cohorts in the incidence of AEs; however, diarrhea and nausea were reported more frequently in patients receiving doses ≥400 vs <400 mg. Anemia and thrombocytopenia were the most frequent grade 3/4 AEs, occurring in seven (16.3%) and 6 (14.0%) patients, respectively. Eight patients discontinued pacritinib because of an AE, three of which were considered to be treatment related (grade 3 prolonged QTc [resolved], grade 3 fatigue [ongoing at the end of follow-up], and grade 3 increased transaminases [resolved]. Dose interruptions or reductions due to AEs occurred in 18 and 9 patients, respectively.

**Table 3:** Treatment-emergent adverse events occurring in ≥10% of patients in phase 1 (*n* = 43)

Treatment-emergent AEs, *n* (%)	100 mg	150 mg	200 mg	300 mg	400 mg	500 mg	600 mg	All patients	
	*n* = 3	*n* = 6	*n* = 9	*n* = 6	*n* = 6	*n* = 7	*n* = 6	*n* = 43	
								Grade 1/2^a^	Grade 3/4^a^
Any AE	3 (100.0)	6 (100.0)	9 (100.0)	6 (100.0)	6 (100.0)	7 (100.0)	6 (100.0)	8 (18.6)	35 (81.4)
Diarrhea	2 (66.7)	2 (33.3)	5 (55.6)	3 (50.0)	6 (100.0)	7 (100.0)	6 (100.0)	28 (65.1)	3 (7.0)
Nausea	0	1 (16.7)	2 (22.2)	3 (50.0)	4 (66.7)	5 (71.4)	4 (66.7)	19 (44.2)	0
Vomiting	0	1 (16.7)	4 (44.4)	2 (33.3)	3 (50.0)	3 (42.9)	3 (50.0)	16 (37.2)	0
Fatigue	1 (33.3)	1 (16.7)	3 (33.3)	2 (33.3)	2 (33.3)	3 (42.9)	1 (16.7)	9 (20.9)	4 (9.3)
Edema peripheral	2 (66.7)	2 (33.3)	1 (11.1)	0	2 (33.3)	2 (28.6)	3 (50.0)	10 (23.3)	2 (4.7)
Pyrexia	1 (33.3)	2 (33.3)	2 (22.2)	1 (16.7)	2 (33.3)	2 (28.6)	2 (33.3)	9 (20.9)	3 (7.0)
Anemia	0	1 (16.7)	1 (11.1)	1 (16.7)	2 (33.3)	2 (28.6)	3 (50.0)	3 (7.0)	7 (16.3)
Constipation	2 (66.7)	2 (33.3)	2 (22.2)	1 (16.7)	2 (33.3)	1 (14.3)	1 (16.7)	11 (25.6)	0
Abdominal distension	0	0	2 (22.2)	1 (16.7)	3 (50.0)	1 (14.3)	2 (33.3)	9 (20.9)	0
Dyspnea	2 (66.7)	1 (16.7)	0	0	2 (33.3)	1 (14.3)	3 (50.0)	9 (20.9)	0
Asthenia	2 (66.7)	2 (33.3)	1 (11.1)	0	0	1 (14.3)	2 (33.3)	6 (14.0)	2 (4.7)
Decreased appetite	2 (66.7)	2 (33.3)	0	0	3 (50.0)	0	1 (16.7)	8 (18.6)	0
Epistaxis	2 (66.7)	1 (16.7)	1 (11.1)	2 (33.3)	1 (16.7)	0	1 (16.7)	7 (16.3)	1 (2.3)
Thrombocytopenia	1 (33.3)	2 (33.3)	0	1 (16.7)	2 (33.3)	1 (14.3)	1 (16.7)	2 (4.7)	6 (14.0)
Abdominal pain	1 (33.3)	1 (16.7)	1 (11.1)	1 (16.7)	2 (33.3)	0	1 (16.7)	6 (14.0)	1 (2.3)
Back pain	1 (33.3)	2 (33.3)	0	1 (16.7)	2 (33.3)	1 (14.3)	0	7 (16.3)	0
Insomnia	1 (33.3)	1 (16.7)	2 (22.2)	1 (16.7)	1 (16.7)	1 (14.3)	0	7 (16.3)	0
Hyperuricemia	1 (33.3)	1 (16.7)	0	0	0	1 (14.3)	3 (50.0)	5 (11.6)	1 (2.3)
Cardiac murmur	1 (33.3)	0	1 (11.1)	0	1 (16.7)	1 (14.3)	2 (33.3)	6 (14.0)	0
Dizziness	0	0	2 (22.2)	1 (16.7)	1 (16.7)	1 (14.3)	1 (16.7)	6 (14.0)	0
Night sweats	1 (33.3)	0	1 (11.1)	0	2 (33.3)	1 (14.3)	1 (16.7)	6 (14.0)	0
Neutropenia	1 (33.3)	2 (33.3)	0	1 (16.7)	1 (16.7)	0	0	2 (4.7)	3 (7.0)
Chills	0	2 (33.3)	0	0	1 (16.7)	1 (14.3)	1 (16.7)	5 (11.6)	0
Cough	1 (33.3)	1 (16.7)	1 (11.1)	1 (16.7)	1 (16.7)	0	0	5 (11.6)	0
Dehydration	0	1 (16.7)	0	0	2 (33.3)	1 (14.3)	1 (16.7)	5 (11.6)	0
Hyperbilirubinemia	1 (33.3)	1 (16.7)	1 (11.1)	1 (16.7)	1 (16.7)	0	0	5 (11.6)	0
Pain in extremity	3 (100.0)	0	0	1 (16.7)	0	0	1 (16.7)	5 (11.6)	0
Petechiae	2 (66.7)	2 (33.3)	0	0	0	1 (14.3)	0	5 (11.6)	0
Skin lesion	0	4 (66.7)	1 (11.1)	0	0	0	0	5 (11.6)	0
Upper respiratory tract infection	0	2 (33.3)	2 (22.2)	1 (16.7)	0	0	0	5 (11.6)	0

^a^Worst grade during treatment

The most frequent AEs considered to be treatment related were diarrhea (65.1%), nausea (34.9%), vomiting (23.3%), and thrombocytopenia (11.6%). Across dose cohorts, larger proportions of patients reported treatment-related AEs in cohorts administered doses ≥400 mg. Grade 3 and 4 treatment-related AEs were reported in nine patients (20.9%) and two patients (4.7%), respectively. No grade 4 gastrointestinal AEs occurred.

SAEs occurred in 19 patients (44.2%) during phase 1 (Additional file 1: Table S2). Four patients (9.3%) experienced a total of five SAEs considered to be treatment related (pleural effusion [two patients; one each in the 100 and 200 mg QD cohorts], tumor lysis syndrome and congestive heart failure [one patient; 400 mg QD cohort], and diarrhea [one patient; 600 mg QD cohort]). All treatment-related SAEs resolved with supportive care. Fourteen patients (32.6%) in phase 1 died during study participation (10 with MF and four with AML). Seven deaths (involving four patients with MF and three with AML) were attributed to AEs (subdural hematoma, intracranial hemorrhage, septic shock, asthenia, cardiorespiratory arrest, anemia, and AML; Additional file 1: Table S2). None of the AEs were considered to be related to treatment. After the 30-day post-treatment discontinuation follow-up, four patients died due to disease progression and three died due to unknown causes.

#### Pharmacokinetics

Pacritinib PK values are shown in Additional file 1: Tables S3 and S4. Median time to maximum concentration (T_max_) values ranging from 4 to 8 h post dose and 2.5 to 5 h post dose were reported on days 1 and 15, respectively. On day 1, mean maximum concentration (C_max_), trough concentration (C_24_) and systemic exposure of pacritinib (AUC_0–24_) generally increased in a dose-related manner; however, increases in exposure appeared to be less than dose-proportional, with systemic exposure appearing to plateau beyond the 400 mg dose level. On day 15, mean C_max_, C_24_ and AUC_0–24_ did not appear to increase in a dose-related manner within the 100–300 mg dose range and, while exposure was higher at the 400 mg dose level relative to the 300 mg dose level, it appeared to plateau above the 400 mg dose level. The between-patient coefficient of variation for C_max_ and AUC_0–24_ ranged from 25 to 63% on day 1 and 20–54% on day 15, indicating relatively high variability across patients.

The mean PK profiles of pacritinib when dosed as citrate and HCl salts were similar (Additional file 1: Figure S1).

#### Efficacy

The clinical benefit rate was 94.4% (34/36) in patients with MF and 42.9% (3/7) in patients with AML, giving an overall clinical benefit rate of 86.0% (13 patients achieving clinical improvement and 24 patients having SD). One patient was reported to have progressive disease, and disease response was not reported for five patients. No clear dose–response correlation was observed.

### Phase 2

#### Patients

Thirty-three patients with MF were enrolled in phase 2, 31 of whom received at least one dose of pacritinib 400 mg QD. Patient demographics and baseline characteristics are shown in Table 4. All patients had splenomegaly with a median length below the left costal margin (as determined by physical examination) of 19 cm (range 7–29). At baseline, grade 2 or higher anemia and thrombocytopenia were reported in 20 (60.6%) and 11 (33.3%) patients, respectively. Six patients (19.4%) were red blood cell (RBC) transfusion dependent at baseline and ten patients (33.3%) had received RBC transfusions within the 180 days prior to study entry. No patients were considered platelet transfusion dependent at baseline, but one patient had received platelet transfusions within 180 days prior to study enrollment. Of the 33 patients enrolled, 13 (39.4%) completed the study and 20 patients (60.6%) discontinued the study prior to completion, primarily because of the sponsor’s decision to terminate the study (*n* = 12) and subject withdrawal of consent (*n* = 5). Of the 31 patients who received at least one dose of pacritinib, median duration of treatment was 11.5 months (range 1.0–20.1). Median actual dose intensity was 371 mg/day (range 208–400) and relative dose intensity was 92.9% (range 52.1–100.0%).

**Table 4:** Baseline characteristics and demographics for patients enrolled in phase 2 (*n* = 33)^a^

	Pacritinib 400 mg
	*n* = 33^b^
Median age, years (range)	67.0 (47–83)
Male, *n* (%)	22 (66.7)
Median time from initial diagnosis, months (range)	31.8 (0.3–210.0)
Median prior systemic therapies, *n* (range)	1 (0–4)
Median time since last MF treatment^c^, months (range)	1.68 (0.6-36.4)
ECOG PS, *n* (%)	
0	6 (18.2)
1	19 (57.6)
2	8 (24.2)
*JAK2* mutation^d^, *n* (%)	27 (81.8)
Type of myelofibrosis, *n* (%)	
Primary myelofibrosis	17 (51.5)
Post-polycythemia vera myelofibrosis	12 (36.4)
Post-essential thrombocythemia myelofibrosis	4 (12.1)
Current Lille Score, *n* (%)	
High risk	1 (3.0)
Intermediate risk	7 (21.2)
Low risk	2 (6.1)
Missing^e^	23 (69.7)
Current DIPSS risk category, *n* (%)	
High risk	7 (21.2)
Intermediate-2 risk	9 (27.3)
Intermediate-1 risk	3 (9.1)
Indeterminate^f^	14 (42.4)
Hemoglobin	
Median^g^, g/L (range)	90.0 (37–144)
Grade 0–2, *n* (%)	27 (81.8)
Grade 3, *n* (%)	6 (18.2)
Baseline hemoglobin level, *n* (%)	
<10 g/L	20 (60.6)
<8 g/L	6 (18.2)
RBC-transfusion dependent^g^, *n* (%)	6 (19.4)
Platelets	
Median^g^, ×10^9^/L (range)	126.0 (28–494)
Grade 0–2, *n* (%)	29 (87.9)
Grade 3, *n* (%)	4 (12.1)
Baseline platelet count, *n* (%)	
<100 × 10^9^/L	10 (42.4)
<50 × 10^9^/L	4 (12.1)
Platelet-transfusion dependent^g^, *n* (%)	0
Leukocytes	
Median^g^, ×10^9^/L (range)	8.91 (1.5–38.2)
Grade 0–2, *n* (%)	31 (93.9)
Grade 3, *n* (%)	2 (6.1)
Baseline leukocyte count >25 × 10^9^/L, *n* (%)	3 (9.1)

^a^Two patients enrolled in the study did not receive the study drug: one patient withdrew consent before beginning treatment and the second patient was unable to undergo magnetic resonance imaging (one of the eligibility criteria)

^b^97% of patients were white

^c^In patients who had received prior treatment (*n* = 25)

^d^All *JAK2* mutations were V617F

^e^Case report forms were designed for direct entry of Lille score by sites; however, some versions of the forms were missing the field for entry of Lille score

^f^Unable to calculate DIPSS risk category due to missing baseline peripheral blood blasts

^g^In the safety population (*n* = 31)

*DIPSS* Dynamic International Prognostic Scoring System, *ECOG PS* Eastern Cooperative Oncology Group Performance Status

#### Efficacy

**Fig. 1:**
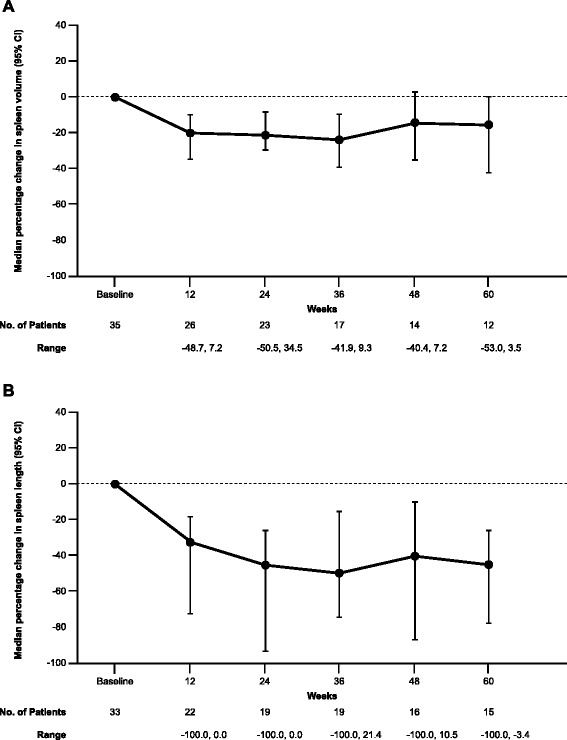
Median percent change in (**a**) spleen volume and (**b**) spleen length from baseline in efficacy evaluable population of the phase 2 study

Spleen responses are summarized for the efficacy evaluable population (Table 5) and displayed as median percent change from baseline up to week 60 (Fig. 1). The primary endpoint (proportion of evaluable patients achieving ≥35% spleen volume reduction at week 24 by MRI) was 23.5% (4/17), with 47.4% (9/19) achieving a ≥50% spleen length reduction at week 24 by physical examination. When assessed up to the end of treatment, 30.4% of patients (7/23) achieved the objective spleen volume reduction and 38.7% (12/31) achieved the objective spleen length reduction. Of the 31 patients who received at least one dose of pacritinib, 14 were not evaluable for spleen response at week 24: 11 discontinued pacritinib prior to week 24 (seven due to lack of response, one due to AEs, and one each due to disease progression, death, and withdrawal of consent) and three remained on pacritinib after week 24, but did not complete the week 24 MRI.

**Table 5:** Summary of best spleen response and symptom score change from baseline in phase 2 (efficacy evaluable population)^a^

Endpoint	Patients achieving endpoint, *n*/*N* (%)			
	95% CI			
	At week 24 visit	Up to week 24 visit	At end of treatment	Up to end of treatment
≥35% spleen volume reduction by MRI	4/17 (23.5)	5/23 (21.7)	4/23 (17.4)	7/23 (30.4)
	95% CI 6.8–49.9		95% CI 5.0–38.8	
≥50% spleen length reduction by physical examination	9/19 (47.4)	12/31 (38.7)	9/31 (29.0)	12/31 (38.7)
	95% CI 24.4–71.1		95% CI 14.2–48.0	
≥50% reduction in MF Quality of Life and Symptom Assessment total symptom score	7/18 (38.9)	12/29 (41.4)	4/29 (13.8)	12/29 (41.4)

^a^Patients included in the efficacy evaluable population are those who had both a non-missing baseline measurement and post-baseline measurement at or through the time point specified

*MRI* magnetic resonance imaging

At week 24, 38.9% of evaluable patients (7/18) had achieved a ≥50% reduction in MF Quality of Life and Symptom Assessment total score relative to baseline (Table 5). Improvement was seen in each of the individual symptoms, as well as the overall quality of life score (Additional file 1: Table S5).

#### Safety

Thirty-one patients were included in the safety population. Table 6 summarizes common treatment-emergent AEs occurring in ≥10% of patients. Gastrointestinal toxicities were reported by all patients. No leukopenia, neutropenia, or lymphopenia was reported during the study. Twenty-two patients (71.0%) reported at least one grade 3/4 AE, most frequently anemia (*n* = 5), diarrhea (5), fatigue (5), and bone pain (4). Grade 3 or 4 thrombocytopenia was reported in three patients. One patient with post-PV MF reported two AEs of pancytopenia, grade 3 and 4 in severity, that were considered unrelated to study drug and resolved. Three patients (9.7%) discontinued pacritinib because of an AE (grade 2 diarrhea, grade 3 diarrhea, and grade 2 anemia). Fourteen patients had dose interruptions and 14 patients had dose reductions because of AEs, most frequently related to diarrhea.

**Table 6:** Treatment-emergent adverse events occurring in ≥10% of patients in phase 2 (*n* = 31)

Treatment-emergent AEs, *n* (%)	Grade 1	Grade 2	Grade 3	Grade 4	Any grade
Diarrhea	14 (45.2)	9 (29.0)	5 (16.1)	0	28 (90.3)
Fatigue	2 (6.5)	11 (35.5)	4 (12.9)	1 (3.2)	18 (58.1)
Nausea	9 (29.0)	6 (19.4)	1 (3.2)	0	16 (51.6)
Edema peripheral	6 (19.4)	5 (16.1)	1 (3.2)	0	12 (38.7)
Vomiting	7 (22.6)	3 (9.7)	1 (3.2)	0	11 (35.5)
Abdominal pain	3 (9.7)	3 (9.7)	3 (9.7)	0	9 (29.0)
Insomnia	7 (22.6)	2 (6.5)	0	0	9 (29.0)
Pruritus	4 (12.9)	4 (12.9)	1 (3.2)	0	9 (29.0)
Pain in extremity	5 (16.1)	2 (6.5)	0	1 (3.2)	8 (25.8)
Night sweats	4 (12.9)	3 (9.7)	0	0	7 (22.6)
Anemia	0	1 (3.2)	3 (9.7)	2 (6.5)	6 (19.4)
Bone pain	1 (3.2)	1 (3.2)	4 (12.9)	0	6 (19.4)
Asthenia	4 (12.9)	1 (3.2)	0	0	5 (16.1)
Constipation	5 (16.1)	0	0	0	5 (16.1)
Dyspnea	4 (12.9)	0	1 (3.2)	0	5 (16.1)
Cardiac murmur	0	4 (12.9)	0	0	4 (12.9)
Hyperuricemia	2 (6.5)	0	0	2 (6.5)	4 (12.9)
Pyrexia	4 (12.9)	0	0	0	4 (12.9)
Rash	2 (6.5)	1 (3.2)	1 (3.2)	0	4 (12.9)
Upper respiratory tract infection	3 (9.7)	1 (3.2)	0	0	4 (12.9)
Weight decreased	4 (12.9)	0	0	0	4 (12.9)

The most frequent AEs considered to be treatment related were diarrhea (*n* = 28; 90.3%), nausea (15; 48.4%), vomiting (9; 29.0%), and fatigue (4; 12.9%). Grade 3 treatment-related AEs were reported in seven patients (22.6%), four of which were diarrhea. No grade 4 treatment-related AEs were reported.

Change over time in hematologic parameters is shown in Additional file 1: Table S6. The percentage change in hemoglobin measurements at each study visit through week 60 relative to baseline remained between a median of −0.5% and +10.1% (range −31% to +146%). At week 24, 10/20 patients (50.0%) had hemoglobin levels <10 g/L and 1/20 patients (5.0%) had hemoglobin levels <8 g/L. During the study, 16 patients (51.6%) received RBC transfusions (median 11 units, range 2–73 units). Platelet counts changed modestly on treatment, with a baseline median platelet count of 126 × 10^9^/L and ranging from a median of 89 to 155 × 10^9^/L through week 60 on study. At week 24, 10/20 patients (50.0%) and 2/20 patients (10.0%) had platelet counts <100 × 10^9^/L and <50 × 10^9^/L, respectively. During the study, three patients (9.7%) received platelet transfusions (median 3 units, range 2–38 units). At week 24, 2/20 patients (10.0%) had leukocyte counts >25 × 10^9^/L. The median neutrophil count was 6.98 x 10^9^/L at baseline and the median ranged from 3.83 to 6.00 × 10^9^/L through week 60 on study.

SAEs occurred in 13 patients (41.9%), two of which were considered to be related to pacritinib (grade 3 diarrhea and grade 3 dehydration; Additional file 1: Table S7). Nine patients (27.3%) who participated in the phase 2 study died, three during the study due to AEs. One patient died from disease progression after the 30-day follow-up and five died due to unknown causes after the 30-day follow-up. None of the deaths were considered related to pacritinib (Additional file 1: Table S7).

## Discussion

This multicenter phase 1/2 study has provided proof-of-concept for selective *JAK2/FLT-3* inhibition with pacritinib as a treatment strategy in MF. Patients treated with pacritinib demonstrated notable reductions in spleen volume and improvements in MF-related symptoms, consistent with findings of another phase 2 study of pacritinib in MF [25] and a phase 3 randomized controlled trial comparing pacritinib with best-available therapy (PERSIST-1) [26, 27]. While the pathogenesis of this disease is complex, it appears that JAK inhibition reduces the inflammatory and proliferative phenotype associated with JAK/STAT-signaling aberrancy [28]. Indeed, the improvements in constitutional symptoms seen with pacritinib in this study are indicative that suppression of *JAK2* even in the absence of *JAK1* blockade is sufficient for inhibition of the inflammatory cytokine pathway responsible for disease symptoms.

In the dose-escalation phase of the study, the MTD was determined to be 500 mg/day, but due to increased tolerability, the recommended daily dose of pacritinib to be taken forward into phase 2 clinical development was 400 mg. This is consistent with the PK profile of pacritinib, where exposure began to plateau at the 400 mg dose.

Pacritinib had an acceptable tolerability profile in this study, with the most common AEs being gastrointestinal disorders, most of which were grade 1 or 2 in severity and resolved with treatment interruptions/dose reductions and supportive care. Gastrointestinal disorders (diarrhea) accounted for treatment discontinuations in only two patients. Of particular interest was the low rate of treatment-emergent anemia or thrombocytopenia during pacritinib treatment, despite no exclusion from participation based on baseline hemoglobin level or platelet count. This is consistent with results from the phase 3 PERSIST-1 study, which showed that pacritinib resulted in durable reductions in spleen volume and symptom burden, including in patients with baseline thrombocytopenia [29].

This study was terminated by the sponsor (S*BIO Pte Ltd, Singapore) for financial reasons in October 2011, meaning that only the data fields assessed as critical for evaluation of efficacy and safety were queried for missing, incorrect or discrepant data prior to database lock. This early termination required all patients who were still receiving pacritinib to stop, limiting our ability to fully assess the long-term effects of pacritinib exposure.

JAK kinase inhibitors have shown the potential to produce durable responses and improve survival in MF [30, 31]; however, minimizing toxicity and avoiding drug resistance are ongoing challenges. Indeed, options remain limited for patients with thrombocytopenia, who are not suitable for treatment with the only currently licensed JAK inhibitor, ruxolitinib. These results suggest that pacritinib has unique characteristics, namely a lack of substantial myelosuppression and manageable non-hematological side effects.

## Conclusions

In this study, pacritinib demonstrated clinical activity in patients with MF that had relapsed or that was poorly controlled with or not suitable for standard therapy. The lack of substantial myelosuppression and manageable side effects seen with pacritinib make it an attractive target for further development in MF.

## Additional files

Additional file 1: Table S1. Dose adjustment and toxicity management guidelines—non-hematologic toxicities. **Table S2** Serious adverse events and deaths^a^ occurring in phase 1 component of the study (*n* = 43). **Table S3** Day 1 pharmacokinetics of pacritinib in the phase 1 part of the study (*n* = 43). **Table S4** Day 15 pharmacokinetics of pacritinib in the phase 1 part of the study *(n* = 43). **Table S5** Percent change from baseline at week 24 for individual and total symptom scores from the Myelofibrosis Quality of Life and Symptom Assessment Tool—phase 2 (efficacy evaluable population). **Table S6** Median percent change from baseline in hematologic parameters over time in the phase 2 component of the study (*n* = 31). **Table S7** Serious adverse events and deaths^a^ occurring in the phase 2 component of the study (*n* = 31). **Figure S1** Mean concentration-time profiles of pacritinib when dosed as hydrochloride and citrate salts at a dose of 200 mg. (DOC 296 kb)

## Abbreviations

AE: Adverse event
AML: Acute myeloid leukemia
C_24_: Trough concentration
C_max_: Maximum concentration
CML: Chronic myeloid leukemia
DLT: Dose-limiting toxicity
ECOG PS: Eastern Cooperative Oncology Group performance status
ET: Essential thrombocythemia
FLT3: FMS-like tyrosine kinase 3
HCl: Hydrochloride
IWG: International Working Group
MF: Myelofibrosis
MPN: Myeloprolifative neoplasm
MRI: Magnetic resonance imaging
MTD: Maximum tolerated dose
PET: Post-essential thrombocythemia
PK: Pharmacokinetic
PPV: Post-polycythemia vera
PV: Polycythemia vera
QD: Once daily
RBC: Red blood cell
SAE: Serious adverse event
SD: Stable disease

## References

[1] Quintas-Cardama A, Verstovsek S (2013). Molecular pathways: Jak/STAT pathway: mutations, inhibitors, and resistance. Clin Cancer Res.

[2] Rawlings JS, Rosler KM, Harrison DA (2004). The JAK/STAT signaling pathway. J Cell Sci.

[3] Baxter EJ, Scott LM, Campbell PJ, East C, Fourouclas N, Swanton S, Vassiliou GS, Bench AJ, Boyd EM, Curtin N, Scott MA, Erber WN, Green AR, Cancer Genome Project (2005). Acquired mutation of the tyrosine kinase JAK2 in human myeloproliferative disorders. Lancet.

[4] Kralovics R, Passamonti F, Buser AS, Teo SS, Tiedt R, Passweg JR, Tichelli A, Cazzola M, Skoda RC (2005). A gain-of-function mutation of JAK2 in myeloproliferative disorders. N Engl J Med.

[5] Levine RL, Wadleigh M, Cools J, Ebert BL, Wernig G, Huntly BJ, Boggon TJ, Wlodarska I, Clark JJ, Moore S, Adelsperger J, Koo S, Lee JC, Gabriel S, Mercher T, D’Andrea A, Frohling S, Dohner K, Marynen P, Vandenberghe P, Mesa RA, Tefferi A, Griffin JD, Eck MJ, Sellers WR, Meyerson M, Golub TR, Lee SJ, Gilliland DG (2005). Activating mutation in the tyrosine kinase JAK2 in polycythemia vera, essential thrombocythemia, and myeloid metaplasia with myelofibrosis. Cancer Cell.

[6] Zhao R, Xing S, Li Z, Fu X, Li Q, Krantz SB, Zhao ZJ (2005). Identification of an acquired JAK2 mutation in polycythemia vera. J Biol Chem.

[7] Pikman Y, Lee BH, Mercher T, McDowell E, Ebert BL, Gozo M, Cuker A, Wernig G, Moore S, Galinsky I, DeAngelo DJ, Clark JJ, Lee SJ, Golub TR, Wadleigh M, Gilliland DG, Levine RL (2006). MPLW515L is a novel somatic activating mutation in myelofibrosis with myeloid metaplasia. PLoS Med.

[8] Klampfl T, Gisslinger H, Harutyunyan AS, Nivarthi H, Rumi E, Milosevic JD, Them NC, Berg T, Gisslinger B, Pietra D, Chen D, Vladimer GI, Bagienski K, Milanesi C, Casetti IC, Sant’Antonio E, Ferretti V, Elena C, Schischlik F, Cleary C, Six M, Schalling M, Schonegger A, Bock C, Malcovati L, Pascutto C, Superti-Furga G, Cazzola M, Kralovics R (2013). Somatic mutations of calreticulin in myeloproliferative neoplasms. N Engl J Med.

[9] Santos FP, Verstovsek S (2012). JAK2 inhibitors for myelofibrosis: why are they effective in patients with and without JAK2V617F mutation?. Anticancer Agents Med Chem.

[10] Frohling S, Schlenk RF, Breitruck J, Benner A, Kreitmeier S, Tobis K, Dohner H, Dohner K, AML Study Group Ulm (2002). Acute myeloid leukemia. Prognostic significance of activating FLT3 mutations in younger adults (16 to 60 years) with acute myeloid leukemia and normal cytogenetics: a study of the AML Study Group Ulm. Blood.

[11] Steudel C, Wermke M, Schaich M, Schakel U, Illmer T, Ehninger G, Thiede C (2003). Comparative analysis of MLL partial tandem duplication and FLT3 internal tandem duplication mutations in 956 adult patients with acute myeloid leukemia. Genes Chromosomes Cancer.

[12] Thiede C, Steudel C, Mohr B, Schaich M, Schakel U, Platzbecker U, Wermke M, Bornhauser M, Ritter M, Neubauer A, Ehninger G, Illmer T (2002). Analysis of FLT3-activating mutations in 979 patients with acute myelogenous leukemia: association with FAB subtypes and identification of subgroups with poor prognosis. Blood.

[13] Desterke C, Bilhou-Nabera C, Guerton B, Martinaud C, Tonetti C, Clay D, Guglielmelli P, Vannucchi A, Bordessoule D, Hasselbalch H, Dupriez B, Benzoubir N, Bourgeade MF, Pierre-Louis O, Lazar V, Vainchenker W, Bennaceur-Griscelli A, Gisslinger H, Giraudier S, Le Bousse-Kerdiles MC, French Intergroup of Myeloproliferative Disorders, French INSERM, European EUMNET Networks on Myelofibrosis (2011). FLT3-mediated p38-MAPK activation participates in the control of megakaryopoiesis in primary myelofibrosis. Cancer Res.

[14] William AD, Lee AC, Blanchard S, Poulsen A, Teo EL, Nagaraj H, Tan E, Chen D, Williams M, Sun ET, Goh KC, Ong WC, Goh SK, Hart S, Jayaraman R, Pasha MK, Ethirajulu K, Wood JM, Dymock BW (2011). Discovery of the macrocycle 11-(2-pyrrolidin-1-yl-ethoxy)-14,19-dioxa-5,7,26-triaza-tetracyclo[19.3.1.1(2,6). 1(8,12)]heptacosa-1(25),2(26),3,5,8,10,12(27),16,21,23-decaene (SB1518), a potent Janus kinase 2/fms-like tyrosine kinase-3 (JAK2/FLT3) inhibitor for the treatment of myelofibrosis and lymphoma. J Med Chem.

[15] Cleary MM, Thompson R, Mahmood S, Davare M, Kurtz S, Elferich J, Shinde U, Druker BJ, Singer J, Agarwal A. Pacritinib, a dual FLT3/JAK2 inhibitor, reduces IRAK-1 signaling in acute myeloid leukemia. Blood. 2015;126(23):Abstract 570.

[16] Singer J, Al-Fayoumi S, Ma H, Komrokji RS, Mesa RA, Verstovsek S (2014). Comprehensive kinase profile of pacritinib, a non-myelosuppressive JAK2 kinase inhibitor in phase 3 development in primary and post ET/PV myelofibrosis. Blood.

[17] Hart S, Goh KC, Novotny-Diermayr V, Hu CY, Hentze H, Tan YC, Madan B, Amalini C, Loh YK, Ong LC, William AD, Lee A, Poulsen A, Jayaraman R, Ong KH, Ethirajulu K, Dymock BW, Wood JW (2011). SB1518, a novel macrocyclic pyrimidine-based JAK2 inhibitor for the treatment of myeloid and lymphoid malignancies. Leukemia.

[18] Hart S, Goh KC, Novotny-Diermayr V, Tan YC, Madan B, Amalini C, Ong LC, Kheng B, Cheong A, Zhou J, Chng WJ, Wood JM (2011). Pacritinib (SB1518), a JAK2/FLT3 inhibitor for the treatment of acute myeloid leukemia. Blood Cancer J.

[19] Dupriez B, Morel P, Demory JL, Lai JL, Simon M, Plantier I, Bauters F (1996). Prognostic factors in agnogenic myeloid metaplasia: a report on 195 cases with a new scoring system. Blood.

[20] Cheson BD, Bennett JM, Kopecky KJ, Buchner T, Willman CL, Estey EH, Schiffer CA, Doehner H, Tallman MS, Lister TA, Lo-Coco F, Willemze R, Biondi A, Hiddemann W, Larson RA, Lowenberg B, Sanz MA, Head DR, Ohno R, Bloomfield CD, International Working Group for Diagnosis, Standardization of Response Criteria, Treatment Outcomes, and Reporting Standards for Therapeutic Trials in Acute Myeloid Leukemia (2003). Revised recommendations of the International Working Group for Diagnosis, Standardization of Response Criteria, Treatment Outcomes, and Reporting Standards for Therapeutic Trials in Acute Myeloid Leukemia. J Clin Oncol.

[21] Cheson BD, Greenberg PL, Bennett JM, Lowenberg B, Wijermans PW, Nimer SD, Pinto A, Beran M, de Witte TM, Stone RM, Mittelman M, Sanz GF, Gore SD, Schiffer CA, Kantarjian H (2006). Clinical application and proposal for modification of the International Working Group (IWG) response criteria in myelodysplasia. Blood.

[22] Tefferi A, Barosi G, Mesa RA, Cervantes F, Deeg HJ, Reilly JT, Verstovsek S, Dupriez B, Silver RT, Odenike O, Cortes J, Wadleigh M, Solberg LA, Camoriano JK, Gisslinger H, Noel P, Thiele J, Vardiman JW, Hoffman R, Cross NC, Gilliland DG, Kantarjian H, IWG for myelofibrosis research and treatment (IWG-MRT) (2006). International Working Group (IWG) consensus criteria for treatment response in myelofibrosis with myeloid metaplasia, for the IWG for myelofibrosis research and treatment (IWG-MRT). Blood.

[23] Cohen MH, Johnson JR, Pazdur R (2005). U.S. Food and Drug Administration Drug Approval Summary: conversion of imatinib mesylate (STI571; Gleevec) tablets from accelerated approval to full approval. Clin Cancer Res.

[24] Mesa RA, Schwager S, Radia D, Cheville A, Hussein K, Niblack J, Pardanani AD, Steensma DP, Litzow MR, Rivera CE, Camoriano J, Verstovsek S, Sloan J, Harrison C, Kantarjian H, Tefferi A (2009). The Myelofibrosis Symptom Assessment Form (MFSAF): an evidence-based brief inventory to measure quality of life and symptomatic response to treatment in myelofibrosis. Leuk Res.

[25] Komrokji RS, Seymour JF, Roberts AW, Wadleigh M, To LB, Scherber R, Turba E, Dorr A, Zhu J, Wang L, Granston T, Campbell MS, Mesa RA (2015). Results of a phase 2 study of pacritinib (SB1518), a JAK2/JAK2(V617F) inhibitor, in patients with myelofibrosis. Blood.

[26] Mesa RA, Egyed M, Szoke A, Suvorov A, Perkins A, Mayer J, Ganly P, Schouten HC, Tosi P, Farber CM, Zachee P, Scheid C, Dean JP, Granston T, Kiladjian JJ, Vannucchi A, Nangalia J, Mead A, Harrison C. Pacritinib (PAC) vs best available therapy (BAT) in myelofibrosis (MF): 60 week follow-up of the phase III PERSIST-1 trial. J Clin Oncol. 2016;34 suppl:Abstract 7065.

[27] Mesa RA, Harrison C, Cervantes F, Dean JP, Wang L, Granston T, Yang Y, Vannucchi A, Mead A. Pacritinib (PAC) vs best available therapy (BAT) in myelofibrosis (MF): long-term follow-up of patient-reported outcomes (PROs) in the phase III PERSIST-1 trial. J Clin Oncol. 2016;34 suppl:Abstract 7067.

[28] Savona MR (2014). Are we altering the natural history of primary myelofibrosis?. Leuk Res.

[29] Harrison CN, Egyed M, Szoke A, Suvorov A, Perkins A, Mayer J, Ganly P, Schouten HC, Tosi P, Farber CM, Zachee P, Scheid C, Dean JP, Zhou H, Kiladjian JJ, Vannucchi A, Nangalia J, Mead A, Mesa RA. Pacritinib (PAC) vs best available therapy (BAT) in myelofibrosis (MF): outcomes in patients (pts) with baseline (BL) thrombocytopenia. J Clin Oncol 2016;34 suppl:Abstract 7011.

[30] Cervantes F, Vannucchi AM, Kiladjian JJ, Al-Ali HK, Sirulnik A, Stalbovskaya V, McQuitty M, Hunter DS, Levy RS, Passamonti F, Barbui T, Barosi G, Harrison CN, Knoops L, Gisslinger H (2013). COMFORT-II Investigators. Three-year efficacy, safety, and survival findings from COMFORT-II, a phase 3 study comparing ruxolitinib with best available therapy for myelofibrosis. Blood.

[31] Verstovsek S, Mesa RA, Gotlib J, Levy RS, Gupta V, DiPersio JF, Catalano JV, Deininger MW, Miller CB, Silver RT, Talpaz M, Winton EF, Harvey JH, Arcasoy MO, Hexner EO, Lyons RM, Raza A, Vaddi K, Sun W, Peng W, Sandor V, Kantarjian H (2015). COMFORT-I Investigators. Efficacy, safety, and survival with ruxolitinib in patients with myelofibrosis: results of a median 3-year follow-up of COMFORT-I. Haematologica.

