# Supplementary material for: Phase 1/2 study of pacritinib, a next generation JAK2/FLT3 inhibitor, in myelofibrosis or other myeloid malignancies
Source: J Hematol Oncol. 2016 Dec 8;9:137. doi: 10.1186/s13045-016-0367-x (PMC5146859; doi:10.1186/s13045-016-0367-x)
Supplement: Additional file 1: Table S1. — Dose adjustment and toxicity management guidelines—non-hematologic toxicities. Table S2 Serious adverse events and deathsa occurring in phase 1 component of the study (n = 43). Table S3 Day 1 pharmacokinetics of pacritinib in the phase 1 part of the study (n = 43). Table S4 Day 15 pharmacokinetics of pacritinib in the phase 1 part of the study (n = 43). Table S5 Percent change from baseline at week 24 for individual and total symptom scores from the Myelofibrosis Quality of Life and Symptom Assessment Tool—phase 2 (efficacy evaluable population). Table S6 Median percent change from baseline in hematologic parameters over time in the phase 2 component of the study (n = 31). Table S7 Serious adverse events and deathsa occurring in the phase 2 component of the study (n = 31). Figure S1 Mean concentration-time profiles of pacritinib when dosed as hydrochloride and citrate salts at a dose of 200 mg. (DOC 296 kb) [file 13045_2016_367_MOESM1_ESM.doc]

# Supplement

**Phase 1/2 study of pacritinib, a next generation JAK2/FLT3 inhibitor, in myelofibrosis or other myeloid malignancies**

**Srdan Verstovsek,**^1^ Olatoyosi Odenike,^2^ Jack W. Singer,^3^ Tanya Granston,^3^ Suliman Al-Fayoumi,^3^ and H. Joachim Deeg^4^

^1^Department of Leukemia, University of Texas MD Anderson Cancer Center, Houston, TX, USA; ^2^Hematology/Oncology, University of Chicago Medical Center, Chicago, IL, USA; ^3^CTI BioPharma Corp., Seattle, WA, USA; and ^4^Fred Hutchinson Cancer Research Center and University of Washington, Seattle, WA, USA.

**Corresponding author**:
Dr. Srdan Verstovsek
Leukemia Department, University of Texas MD Anderson Cancer Center, 1515 Holcombe Blvd, Unit 428, Houston, TX 77030, USA
Phone: +1-713-745-3429
Fax: +1-713-745-0390
Email: [sverstov@mdanderson.org](mailto:sverstov@mdanderson.org)

Supplemental Table 1. Dose adjustment and toxicity management guidelines – non-hematologic toxicities.

| **Event** | **Grade** | **Management/action** |
| --- | --- | --- |
| Any non-hematologic event that was related to the study drug | 1 or 2 | No change |
|  | 3 | Hold treatment. Treatment may resume if toxicity is resolved to grade ≤1 or returned to baseline. Starting dose may be at the same level if the toxicity resolves within 7 days or decreased by 25% daily at the discretion of the investigator after discussion with the sponsor. Toxicity lasting >7 days requires doses reduction by ≥25% daily at the discretion of the investigator after discussion with the sponsor. Dose reductions were accomplished using available strengths of the study drug, rounding down if necessary. |
|  | 4 | Hold treatment. Treatment may resume if toxicity is resolved to grade ≤1 or returned to baseline at the discretion of the investigator after discussion with the sponsor. A 25% daily dose decrease is required at treatment resumption. Dose reductions were accomplished using available strengths of the study drug, rounding down if necessary. |

**Supplemental Table 2. Serious adverse events and deaths^a^ occurring in phase 1 component of the study (n = 43)**

| **Patient** | **Malignancy type** | **SAE** | **Grade** | **Causality** | **Management/Outcome** |
| --- | --- | --- | --- | --- | --- |
| 1. | MF | Pleural effusion | 3 | Possibly related | Drug interrupted; recovered/resolved |
|  |  | Pneumonia | 3 | Not related | Dose not changed; recovered/resolved with sequelae |
|  |  | Neutropenia | 3 | Not related | Drug interrupted; recovered/resolved |
|  |  | Subacute left subdural hematoma | 4 | Not related | Drug withdrawn; recovered/resolved |
| 2. | MF | Stroke | 4 | Not related | Drug withdrawn; recovered/resolved with sequelae |
| 3. [Death on study] | AML | Subdural hematoma | 3 | Not related | Drug interrupted; recovered/resolved with sequelae |
|  |  | Abdominal pain | 2 | Not related | Dose not changed; recovered/resolved |
|  |  | Subdural hematoma | 5 | Not related | Fatal |
| 4. | MF | Fever | 3 | Not related | Dose not changed; recovered/resolved |
| 5. | MF | Acute renal failure | 3 | Not related | Drug interrupted; recovered/resolved |
| 6. | MF | Bronchitis infection with normal ANC | 3 | Not related | Drug interrupted; recovered/resolved |
|  |  | Fever | 2 | Not related | Dose not changed; recovered/resolved |
|  |  | Bilateral wrist pain | 2 | Not related | Dose not changed; recovered/resolved |
| 7. | MF | Chest pain | 3 | Not related | Dose not changed; recovered/resolved |
| 8. | MF | Syncope | 3 | Not related | Drug interrupted; recovered/resolved |
|  |  | Syncope | 3 | Not related | Drug interrupted; recovered/resolved |
|  |  | *C. difficile* colitis | 3 | Not related | Dose not changed; recovered/resolved |
|  |  | Diarrhea | 3 | Possibly related | Drug interrupted; recovered/resolved |
| 9. [Death on study] | MF | Transitional cell bladder cancer | 4 | Not related | Dose not changed; recovered/resolved |
|  |  | Septic shock | 5 | Not related | Fatal |
| 10. [Death on study] | MF | Pneumonia | 3 | Not related | Dose not changed; recovered/resolved |
|  |  | Asthenia | 5 | Not related | Fatal |
| 11. | MF | Anemia | 4 | Not related | Dose not changed; recovered/resolved |
| 12. [Death on study] | AML | Bacterial infection | 3 | Not related | Dose not changed; recovered/resolved |
|  |  | Cardiorespiratory arrest | 5 | Not related | Fatal |
| 13. | MF | Pneumonia | 3 | Not related | Drug interrupted; recovered/resolved |
|  |  | Right-sided heart failure | 3 | Not related | Drug interrupted; recovered/resolved |
| 14. [Death on study] | MF | Intracranial bleed | 5 | Not related | Fatal |
| 15. | MF | Abscess requiring incision/drainage | 3 | Not related | Drug interrupted; recovered/resolved |
| 16. | MF | Left pleural effusion | 2 | Possibly related | Dose not changed; recovered/resolved |
| 17. | MF | Sepsis | 3 | Not related | Drug interrupted; recovered/resolved |
| 18. [Death on study] | MF | Severe anemia | 5 | Fatal |  |
| 19. [Death on study] | AML | Tumor lysis | 3 | Probably related | Dose not changed; recovered/resolved |
|  |  | Congestive heart failure | 3 | Possibly related | Dose not changed; recovered/resolved |
|  |  | Deteriorating AML | 5 | Not related | Fatal |

1. Note that four patients died due to disease progression and three patients died due to unknown causes after the 30-day post-treatment discontinuation follow-up period.

Supplemental Table 3. Day 1 pharmacokinetics of pacritinib in the phase 1 part of the study (n = 43).

| Parameter | Pacritinib | | | | | | |
| --- | --- | --- | --- | --- | --- | --- | --- |
|  | 100 mg | 150 mg | 200 mg^a^ | 300 mg | 400 mg | 500 mg | 600 mg |
| C_max_ |  |  |  |  |  |  |  |
| N | 3 | 6 | 9 | 6 | 6 | 7 | 6 |
| Mean | 3699 | 3723 | 4999 | 5235 | 6275 | 7052 | 8329 |
| % CV | 45.8 | 48.6 | 28.0 | 39.4 | 62.6 | 55.7 | 24.8 |
| Median | 2906 | 3794 | 4571 | 5833 | 5644 | 6310 | 7877 |
| Range | 2549-5644 | 1667-6244 | 3153­­-7775 | 2116-7588 | 2023-13422 | 1211-12655 | 5981-11301 |
| T_max_ |  |  |  |  |  |  |  |
| N | 2 | 3 | 6 | 4 | 6 | 3 | 3 |
| Median | 5 | 4 | 5 | 4 | 8 | 4 | 4.5 |
| Range | 3-5 | 3-24 | 3-24 | 3-24 | 5-24 | 3-8 | 2-5 |
| AUC_0-24h_ |  |  |  |  |  |  |  |
| N | 3 | 6 | 9 | 6 | 6 | 7 | 6 |
| Mean | 65931 | 64717 | 93283 | 87121 | 126608 | 141152 | 135831 |
| % CV | 40.8 | 39.0 | 26.6 | 54.2 | 60.9 | 60.6 | 47.6 |
| Median | 56034 | 69156 | 87286 | 98217 | 117611 | 117821 | 128781 |
| Range | 45369-96390 | 34140-93657 | 61055-137843 | 4353-142136 | 35546-262182 | 22114-264974 | 41880-221135 |
| C_24h_ |  |  |  |  |  |  |  |
| N | 3 | 6 | 9 | 6 | 6 | 7 | 6 |
| Mean | 2440 | 3113 | 3921 | 4213 | 5644 | 5899 | 5093 |
| % CV | 36.5 | 66.5 | 45.6 | 46.7 | 73.5 | 67.8 | 48.4 |
| Median | 2318 | 2316 | 3026 | 3528 | 4725 | 4417 | 4136 |
| Range | 1617-3386 | 1308-6244 | 2254-7775 | 2482-7588 | 1096-13422 | 789-11736 | 2768-8193 |

1. Cohorts for the pacritinib HCl salt formulation and citrate salt formulation were pooled, because they were at the same dose. The two salt forms have been shown to be pharmaceutically equivalent.

AUC_0-24h_ = area under the concentration-time curve from time zero to 24 hours; C_max_ = maximum concentration; C_24h_ = concentration at 24 hours; %CV = percent coefficient of variation; T_max_ = time to maximum concentration.

Supplemental Table 4. Day 15 pharmacokinetics of pacritinib in the phase 1 part of the study (n = 43).

| Parameter | Pacritinib | | | | | | |
| --- | --- | --- | --- | --- | --- | --- | --- |
|  | 100 mg | 150 mg | 200 mg^a^ | 300 mg | 400 mg | 500 mg | 600 mg |
| C_max_ |  |  |  |  |  |  |  |
| N | 3 | 6 | 9 | 6 | 6 | 7 | 6 |
| Mean | 6362 | 6698 | 6696 | 6407 | 9770 | 8726 | 9225 |
| % CV | 24.4 | 48.0 | 27.9 | 33.4 | 49.2 | 43.2 | 20.0 |
| Median | 6181 | 5411 | 6454 | 6855.5 | 11677.5 | 7986 | 8555 |
| Range | 4910-7996 | 3543-11236 | 2878-9227 | 2444-8726 | 2712-14820 | 2771-14333 | 7707-12714 |
| T_max_ |  |  |  |  |  |  |  |
| N | 2 | 4 | 5 | 4 | 6 | 3 | 3 |
| Median | 3 | 2.5 | 5 | 4 | 341 | 4 | 4 |
| Range | 3-4 | 2-24 | 0.5-8 | 2-5 | 338-360 | 3-6 | 4-8 |
| AUC_0-24h_ |  |  |  |  |  |  |  |
| N | 3 | 6 | 9 | 6 | 6 | 7 | 6 |
| Mean | 120306 | 139901 | 125115 | 132905 | 202878 | 178367 | 161277 |
| % CV | 26.3 | 53.8 | 35.7 | 37.8 | 51.2 | 49.1 | 45.2 |
| Median | 121364 | 114158.5 | 130082 | 133880 | 239919 | 160870 | 160969 |
| Range | 88135-151420 | 59673-235826 | 44132-171545 | 45528-194622 | 54178- 318092 | 58206- 323382 | 48272-276093 |
| C_24h_ |  |  |  |  |  |  |  |
| N | 3 | 6 | 9 | 6 | 6 | 7 | 6 |
| Mean | 3945 | 5428 | 4852 | 4965 | 8429 | 6550 | 6439 |
| % CV | 24.4 | 55.6 | 26.5 | 46.1 | 57.4 | 58.2 | 37.4 |
| Median | 4322 | 5026 | 4993 | 4850 | 10168 | 5685 | 5417 |
| Range | 2849-4663 | 1973-9277 | 2427-6187 | 1421-8302 | 1713-12776 | 1994-12908 | 4410-10256 |

1. Cohorts for the pacritinib HCl salt formulation and citrate salt formulation were pooled, because they were at the same dose. The two salt forms have been shown to be pharmaceutically equivalent.

AUC_0-24h_ = area under the concentration-time curve from time zero to 24 hours; C_max_= maximum concentration; C_24h_ = concentration at 24 hours; %CV = percent coefficient of variation; T_max_ = time to maximum concentration.

**Supplemental Table 5. Percent change from baseline at Week 24 for individual and total symptom scores from the Myelofibrosis Quality of Life and Symptom Assessment Tool - phase 2 (efficacy evaluable population).**

| Symptom | N | Mean percent change from baseline at Week 24 (SD) |
| --- | --- | --- |
| Fatigue – worst | 18 | -12.5 (63.55) |
| Early satiety | 17 | -42.2 (37.53) |
| Abdominal pain | 16 | -54.2 (58.14) |
| Night sweats | 11 | -31.3 (85.45) |
| Itching (pruritus) | 12 | -62.6 (45.35) |
| Bone pain | 11 | -51.6 (43.04) |
| Overall quality of life | 16 | -38.1 (49.50) |
| Total symptom score**^a^** | 18 | -27.5 (39.01) |

1. Total symptom score is the sum of the individual scores of worst fatigue, early satiety, abdominal pain or discomfort, night sweats, itching (pruritus), and bone pain.

**Supplemental Table 6. Median percent change from baseline in hematologic parameters over time in phase 2 component of the study (n = 31)**

| **Parameter (% change from baseline)^a^** | **Week** | | | | | | | | | | | | |
| --- | --- | --- | --- | --- | --- | --- | --- | --- | --- | --- | --- | --- | --- |
|  | **2** | **4** | **6** | **8** | **10** | **12** | **16** | **20** | **24** | **36** | **48** | **60** | **Termi-nation** |
| N | 28 | 31 | 24 | 28 | 22 | 24 | 23 | 20 | 20 | 20 | 17 | 15 | 8 |
| Platelets |  |  |  |  |  |  |  |  |  |  |  |  |  |
| Median | -1.6 | -20.3 | -3.6 | -4.5 | -3.1 | -7.7 | -20.1 | -11.7 | -23.7 | -23.4 | -16.3 | -31.3 | -5.4 |
| Range | -45, 75 | -56, 74 | -47, 103 | -66, 88 | -53, 98 | -24.1, 4.3 | -48, 94 | -48, 27 | -51, 48 | -67, 70 | -81, 93 | -70, 108 | -52, 3 |
| Red blood cells | |  |  |  |  |  |  |  |  |  |  |  |  |
| Median | 2.23 | 1.41 | -1.65 | 2.14 | -1.37 | -1.81 | 6.16 | -0.30 | 2.46 | -3.15 | -3.87 | 1.48 | 0.18 |
| Range | -12.5, 78.3 | -12.2, 29.4 | -17.8, 31.3 | -28.1, 32.3 | -24.1, 85.6 | -21.2, 46.6 | -20.8, 53.6 | -27.1, 30.9 | -26.9, 42.3 | -22.9, 34.0 | -20.3, 44.8 | -26.9, 25.3 | -23.6, 41.0 |
| Leukocytes |  |  |  |  |  |  |  |  |  |  |  |  |  |
| Median | -5.93 | -26.77 | -5.58 | -5.50 | 7.90 | -21.83 | -15.09 | -25.83 | -30.34 | -18.32 | -5.13 | -28.21 | 15.49 |
| Range | -53.1, 42.8 | -83.8, 102.7 | -57.7, 78.2 | -58.6, 124.7 | -42.9, 173.3 | -60.3, 91.7 | -32.8, 56.1 | -55.8, 86.5 | -63.8, 144.0 | -75.0, 102.7 | -66.0, 232.0 | -71.8, 292.0 | -65.7, 164.5 |
| Neutrophils |  |  |  |  |  |  |  |  |  |  |  |  |  |
| Median | -16.44 | -26.43 | -9.32 | -19.76 | -1.44^b^ | -31.39 | -33.04 | -32.93 | -43.24 | -33.96 | -28.84 | -40.17 | -9.19 |
| Range | -61.6, 54.9 | -76.5, 134.8 | -72.0, 106.8 | -60.3, 180.0 | -77.8, 183.7 | -73.2, 145.6 | -64.2, 100.8 | -58.0, 174.4 | -65.4, 137.5 | -75.6, 97.2 | -74.6, 231.7 | -76.5, 281.5 | -70.6, 229.7 |
| Lymphocytes |  |  |  |  |  |  |  |  |  |  |  |  |  |
| Median | 26.93 | -6.84 | 31.7 | 12.65 | 52.34 | 20.88 | 22.22 | 37.07 | 44.14 | 58.89 | 61.70 | 40.43 | 39.87 |
| Range | -53.2, 184.9 | -97.8, 419.0 | -72.9, 273.0 | -54.7, 283.0 | -93.4, 1338.0 | -72.9, 194.4 | -53.3, 212.0 | -60.5, 250.9 | -68.9, 261.1 | -54.3, 238.9 | -65.6, 156.5 | -43.5, 213.0 | -56.0, 213.0 |
| Hemoglobin |  |  |  |  |  |  |  |  |  |  |  |  |  |
| Median | 5.3 | 2.2 | 9.1 | 4.5 | 8.2 | -0.5 | 8.6 | 5.8 | 7.3 | -0.4 | 10.1 | 4.7 | 5.0 |
| Range | -14, 146 | -17, 146 | -17, 141 | -30, 119 | -15, 116 | -24, 116 | -20, 138 | -30, 81 | -31, 34 | -24, 34 | -18, 32 | -21, 37 | -21, 132 |
| Hematocrit |  |  |  |  |  |  |  |  |  |  |  |  |  |
| Median | 2.92 | 0.00 | 0.64 | 2.01 | -3.57 | -3.31 | 3.11 | -1.30 | 3.01 | -2.91 | -2.39 | 3.70 | 0.48 |
| Range | -12.82, 56.58 | -16.62, 22.50 | -19.41, 27.75 | -30.65, 27.90 | -22.59, 57.60 | -21.89, 38.64 | -22.58, 40.55 | -29.49, 20.74 | -28.34, 32.72 | -23.04, 29.95 | -19.35, 37.12 | -23.27, 28.99 | -20.97, 37.24 |

1. Excludes patients with a baseline value of 0.
2. n = 19.

**Supplemental Table 7. Serious adverse events and deaths^a^ occurring in phase 2 component of the study (n = 31)**

| **Patient** | **SAE** | **Grade** | **Causality** | **Management/Outcome** |
| --- | --- | --- | --- | --- |
| 1. | Diarrhea | 3 | Related | Grade 3 diarrhea developed on Day 18 of the study. Diarrhea resolved within 4 days and the subject was able to continue pacritinib at 400 mg for a total of 595 days. |
| 2. | Dehydration | 3 | Related | Grade 3 dehydration developed on Day 17 of the study in the context of fever, nausea, and vomiting. The subject was treated with transfusions, antibiotics, antiemetics, and antidiarrheals, while pacritinib was interrupted for 1 day. Pacritinib was restarted and the subject remained on treatment for a total of 607 days. |
|  | Back pain | 3 | Not related | Dose not changed; recovered/resolved |
|  | Chest pain | 3 | Not related | Drug interrupted; recovered/resolved |
|  | Urinary tract infection | 3 | Not related | Dose not changed; recovered/resolved |
|  | Altered mental status | 3 | Not related | Drug interrupted; recovered/resolved |
| 3. | Pulmonary hypertension | 3 | Not related | Dose not changed; recovered/resolved with sequelae |
| 4. [Death on study] | Failure to thrive | 4 | Not related | Dose not changed; recovered/resolved with sequelae |
|  | Hematemesis | 3 | Not related | Dose not changed; recovered/resolved |
|  | Cachexia | 5 | Not related | A 76-year old man with primary MF and a history of prostate cancer, mucosal-associated lymphoid tissue lymphoma and coronary artery disease discontinued pacritinib due to lack of response on Day 113 and his MF was subsequently treated with prednisone and danazol. He presented on Day 132 with hematemesis, weakness, cachexia and a markedly enlarged spleen. Because of his clinical deterioration, his MF therapies were discontinued and he died 4 days later due to cachexia from progressive MF. |
| 5. [Death during 30-day follow-up] | Infection – abdominal abscess | 3 | Not related | Dose not changed; recovered/resolved |
|  | Myelofibrosis | 5 | Not related | Fatal |
| 6. | Splenomegaly | 3 | Not related | Dose not changed; recovered/resolved |
|  | Tracheobronchitis | 3 | Not related | Dose not changed; recovered/resolved with sequelae |
|  | Intra-abdominal hematoma | 3 | Not related | Dose not changed; recovered/resolved |
| 7. | Atrial fibrillation with rapid ventricular rate | 3 | Not related | Drug interrupted; recovered/resolved with sequalae |
|  | Congestive heart failure | 3 | Not related | Dose not changed; recovered/resolved |
| 8. [Death on study] | Incarcerated umbilical hernia | 3 | Not related | Drug interrupted; recovered/resolved |
|  | Gram-negative sepsis | 5 | Not related | A 67-year old man with primary MF died after he developed renal failure in the setting of severe gram-negative septicemia. The subject had undergone surgery for an incarcerated umbilical hernia 4 months prior and had portal hypertension and ascites, requiring frequent therapeutic paracenteses |
| 9. | Infection – pneumonia | 3 | Not related | Dose not changed; recovered/resolved |
|  | Anemia | 2 | Not related | Drug interrupted; recovered/resolved |
|  | Pain due to peripheral neuropathy | 3 | Not related | Dose not changed; recovered/resolved |
|  | Pancytopenia | 4 | Not related | Dose not changed; recovered/resolved |
|  | Exacerbation of pain due to peripheral neuropathy | 3 | Not related | Dose not changed; recovered/resolved |
|  | Pancytopenia | 3 | Not related | Dose not changed; recovered/resolved |
|  | Congestive heart failure | 3 | Not related | Dose not changed; recovered/resolved |
|  | Hemoptysis | 3 | Not related | Dose not changed; recovered/resolved |
| 10. | Anemia worsening | 3 | Not related | Drug interrupted; recovered/resolved |
|  | Subdural hematoma | 3 | Not related | Dose not changed; recovered/resolved |
|  | Pericarditis | 3 | Not related | Dose not changed; recovered/resolved |
|  | Epistaxis | 3 | Not related | Dose not changed; recovered/resolved |
|  | Right lower lobe pneumonia | 3 | Not related | Dose not changed; recovered/resolved |
| 11. | Complete heart block | 3 | Not related | Drug interrupted; recovered/resolved |
| 12. | Exacerbation of congestive heart failure (x 3) | 3 | Not related | Dose not changed; recovered/resolved |
|  | Cardiac ischemia | 3 | Not related | Dose not changed; recovered/resolved |
|  | Splenic infarction | 3 | Not related | Dose not changed; recovered/resolved |
|  | ST-elevation myocardial infarction | 3 | Not related | Dose not changed; recovered/resolved |
| 13. [Death on study] | Lower extremity pain | 4 | Not related | Drug interrupted; recovered/resolved |
|  | Severe edema, lower extremity | 3 | Not related | Dose not changed; recovered/resolved |
|  | Hemorrhage | 5 | Not related | A 74-year old woman with PPV-MF and portal hypertension sustained a fatal arterial laceration during a therapeutic paracentesis. |

1. Note that five patients died due to unknown causes after the 30-day post-treatment discontinuation follow-up period.

**Supplemental Figure 1. Mean concentration-time profiles of pacritinib when dosed as hydrochloride and citrate salts at a dose of 200 mg**


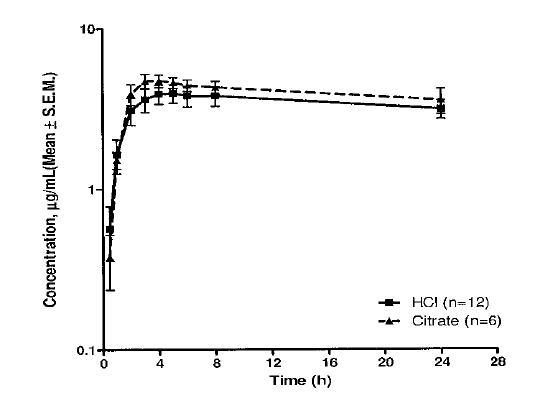


The hydrochloride salt curve was obtained by pooling the concentrations from 12 patients entrolled in the 200 mg cohorts of three pacritinib studies (NCT00719836, NCT00741871 and NCT00745550]). The citrate salt curve is the mean concentration from six patients enrolled in the 200 mg cohort of this present study (NCT00719836).
